# Dual processing of sulfated steroids in the olfactory system of an anuran amphibian

**DOI:** 10.3389/fncel.2015.00373

**Published:** 2015-09-23

**Authors:** Alfredo Sansone, Thomas Hassenklöver, Thomas Offner, Xiaoyan Fu, Timothy E. Holy, Ivan Manzini

**Affiliations:** ^1^Institute of Neurophysiology and Cellular Biophysics, University of GöttingenGöttingen, Germany; ^2^Center for Nanoscale Microscopy and Molecular Physiology of the BrainGöttingen, Germany; ^3^Department of Anatomy and Neurobiology, Washington University School of MedicineSt. Louis, MO, USA

**Keywords:** intraspecific chemical communication, *Xenopus laevis*, pipidae, main olfactory system, vomeronasal system

## Abstract

Chemical communication is widespread in amphibians, but if compared to later diverging tetrapods the available functional data is limited. The existing information on the vomeronasal system of anurans is particularly sparse. Amphibians represent a transitional stage in the evolution of the olfactory system. Most species have anatomically separated main and vomeronasal systems, but recent studies have shown that in anurans their molecular separation is still underway. Sulfated steroids function as migratory pheromones in lamprey and have recently been identified as natural vomeronasal stimuli in rodents. Here we identified sulfated steroids as the first known class of vomeronasal stimuli in the amphibian *Xenopus laevis*. We show that sulfated steroids are detected and concurrently processed by the two distinct olfactory subsystems of larval *Xenopus laevis*, the main olfactory system and the vomeronasal system. Our data revealed a similar but partially different processing of steroid-induced responses in the two systems. Differences of detection thresholds suggest that the two information channels are not just redundant, but rather signal different information. Furthermore, we found that larval and adult animals excrete multiple sulfated compounds with physical properties consistent with sulfated steroids. Breeding tadpole and frog water including these compounds activated a large subset of sensory neurons that also responded to synthetic steroids, showing that sulfated steroids are likely to convey intraspecific information. Our findings indicate that sulfated steroids are conserved vomeronasal stimuli functioning in phylogenetically distant classes of tetrapods living in aquatic and terrestrial habitats.

## Introduction

The mammalian olfactory system is typically defined by at least two anatomically and molecularly segregated subsystems, the most prominent being the main and vomeronasal system. The main olfactory system generally senses volatile chemicals primarily signaling food and environmental cues, whereas the vomeronasal system mainly detects pheromones, which signal intraspecific social cues (Liberles, 2014). Several recent studies, however, showed that this separation may not be as strict as originally thought (Zufall and Leinders-Zufall, 2007). Fishes generally have a single olfactory system. Characteristic morphological sensory neuron types, as well as genetic components of both the mammalian main olfactory epithelium (MOE) and vomeronasal organ (VNO) coexist in their unique olfactory epithelium. Accordingly, it mediates all of the behavioral responses known to be associated with the sense of smell (Hamdani and Døving, 2007).

Amphibians are transitional species, typically having aquatic larvae and terrestrial or secondarily aquatic adults. Most amphibians, including *Xenopus laevis* (see Dittrich et al., 2015), have anatomically segregated main and vomeronasal systems (Eisthen and Polese, 2006). However, at a cellular and molecular level the systems are not yet fully separated. The MOE of larval *Xenopus* still closely resembles the single olfactory epithelium of fishes. It is made of ciliated as well as microvillous sensory neurons expressing olfactory receptor genes of all main families (Hansen et al., 1998; Hagino-Yamagishi et al., 2004; Date-Ito et al., 2008; Gliem et al., 2013; Syed et al., 2013). Also, at least two different transduction pathways are present (Gliem et al., 2013; Sansone et al., 2014a,b). Conversely, the larval VNO is already very similar to that of rodents, comprising microvillous neurons (Hansen et al., 1998), which express solely vomeronasal type-2 receptor genes (Hagino-Yamagishi et al., 2004; Syed et al., 2013; Sansone et al., 2014b).

Many general mammalian odorants, mostly volatile chemicals, have been identified. The number of known mammalian pheromones, many of them urinal constituents, is much lower (Liberles, 2014). Sulfated steroids have been reported to be the predominant vomeronasal ligands in female mouse urine (Nodari et al., 2008). The olfactory system of fishes detects general odorants and substances with pheromonal activity (Bazáes et al., 2013). Some sulfated steroids function as migratory pheromones in lamprey (Sorensen et al., 2005). In aquatic amphibians (larval and adult) the main olfactory system is generally sensitive to the same odorant classes as in fishes. Terrestrial species typically sense the same odorants as mammals (Eisthen and Polese, 2006). Pheromonal communication is relatively widespread in caudate amphibians, however, much less is known in anurans, where acoustic communication is thought to be the main mode of intraspecific communication (Woodley, 2014).

Here we report that the same synthetic sulfated steroids active in rodents, elicit strong olfactory responses in both the main and vomeronasal system of larval *Xenopus laevis*. Sulfated steroids thus appear to be conserved vomeronasal stimuli in phylogenetically distant classes of tetrapods. Analysis of neuronal responses revealed similarities and differences in the processing of these stimuli in the two subsystems. Furthermore, mass spectrometry analysis revealed multiple sulfated compounds in *Xenopus* excretions with mass-to-charge ratios consistent with sulfated steroids. The fact that these excretion products activated a substantial subset of sensory neurons responsive to the synthetic compounds indicates that sulfated metabolites are involved in amphibian intraspecific communication.

## Materials and methods

### Confocal Ca^2+^ imaging in acute slices of the olfactory organ

Slices (130–150 μm) of the olfactory organ of larval *Xenopus laevis* (either sex, stages 50–54; see ref. Nieuwkoop and Faber, 1994) were prepared and loaded with Fluo-4 AM (Life Technologies) as described in earlier work of our lab (Sansone et al., 2014a). All procedures for animal handling were approved by the governmental animal care and use office (Niedersächsisches Landesamt für Verbraucherschutz und Lebensmittelsicherheit, Oldenburg, Germany, Protocol No. T24.07) and were in accordance with the German Animal Welfare Act as well as with the guidelines of the Göttingen University Committee for Ethics in Animal Experimentation. Reproducibility of stimulus-induced responses was verified by regularly repeating the application at least twice. As a negative control we regularly applied standard bath solution containing the same DMSO concentration as the stimuli. The minimum interstimulus interval was at least 2 min. Changes of intracellular calcium concentrations were monitored at 1 Hz using an inverted or upright laser-scanning confocal microscope (LSM 510/Axiovert 100 M or LSM 780/Axio Examiner, Carl Zeiss).

### Multi-photon Ca^2+^ imaging in the whole mount olfactory bulb

To image activity in the olfactory bulb a bolus injection of Fluo-4 AM was performed. For orientation during this procedure we previously electroporated neurons in the MOE and VNO with Alexa-594 dextran (for details see Hassenklöver and Manzini, 2013, 2014). After killing the animals the connective tissue covering the ventral side of the telencephalon was removed using fine scissors and the preparation was mechanically fixed in a recording chamber and covered with bath solution. A solution containing a saturated solution of Fluo-4 AM was filtered, centrifuged, and then the supernatant was used to fill borosilicate pipettes (resistance ~10–15 MΩ). The pipette, controlled via a micromanipulator, was carefully introduced into the olfactory bulb in close proximity of the axon terminals and the Fluo-4 AM solution was ejected by pressure application. After 45 min incubation at room temperature, post-synaptic Ca^2+^-responses were imaged using a multi-photon microscope (Nikon A1R-MP, Nikon). Fluorescence image stacks containing glomeruli and neurons of the main and accessory olfactory bulb were acquired with a fast resonant scanning unit. Image stacks were acquired at 0.7–1 Hz with an interval of 3–5 μm between the acquired planes.

### Analysis of Ca^2+^ imaging data

Fluorescence changes for individual regions of interest, i.e., individual neurons or glomeruli, are given as ΔF/F values. The fluorescence changes were calculated as ΔF/F = (F–F_0_)/F_0_, where F was the fluorescence averaged over the pixels of a sensory neuron, while F_0_ was the average fluorescence of that sensory neuron prior to stimulus application. A response was assumed if the following criteria were met: (i) the maximum amplitude of the calcium transient had to be higher than the maximum of the prestimulus intensities; (ii) the onset of the response had to be within 10 frames after stimulus application. The analysis of confocal imaging data was carried out as described in earlier work of our lab (Sansone et al., 2014a). For dose-response analysis, responses to each concentration were pooled and fitted using GraphPad Prism (GraphPad Software). Detection threshold for every cell was determined as the lowest concentration at which a response meeting above criteria occurred. The overlaps of reactivity between the single components of sulfated steroid mixtures were visualized as area-weighted Venn diagrams created with Python/matplotlib-venn. To determine reactive regions in the multi-photon imaging data, difference image stacks were calculated by subtracting the mean background activity before stimulus application from the mean peak response. Difference image stacks were converted to binary data by adaptive thresholding. Mean traces of calcium transients were extracted from volumetric regions of interest derived from these binary image stacks. For visualization, the binary data was smoothed and rendered as a 3D volume. All the Ca^2+^ imaging data were analyzed using custom-written programs in MATLAB (Mathworks, Natick, USA).

### Olfactory stimuli and solutions

All the stimuli were dissolved in bath solution or DMSO according to the specific solubility and were applied at a final concentration of 100–200 μM (additional dilutions were used in dose-response experiments). The stimuli used were: sulfated steroids (Steraloids, see Supplementary Table 1), non-sulfated steroids (β-estradiol, pregnanolone, allopregnanolone, progesterone; Sigma), a mixture of 19 amino acids (Sigma; Manzini and Schild, 2004), tadpole and adult frog breeding water (diluted 1:1 in bath solution). Sulfated steroids were applied as mixtures in some of the experiments (E mix: E0588, E1050, E2734; P mix: P3817, P8168, P8200). Amino acids are well characterized odorants in *Xenopus laevis* (Manzini and Schild, 2004; Gliem et al., 2013; Sansone et al., 2014a). To collect excretion products, we kept either 80–100 tadpoles or 10–20 post-metamorphic frogs in a beaker filled with 500 ml tap water for 12–24 h. After removing the animals, the water was filtered with a Falcon cell strainer (40 μm nylon mesh; BD Biosciences) to remove particles and stored at +4°C. Bath solution consisted of (in mM): 98 NaCl, 2 KCl, 1 CaCl_2_, 2 MgCl_2_, 5 glucose, 5 Na-pyruvate, 10 HEPES. The composition of the Ca^2+^-free bath solution was (in mM): 98 NaCl, 2 KCl, 2 MgCl_2_, 5 glucose, 5 Na-pyruvate, 10 HEPES, 2 EGTA. High K^+^ bath solution consisted of (in mM): 17 NaCl, 80 KCl, 1 CaCl_2_, 2 MgCl_2_, 5 glucose, 5 Na-pyruvate, 10 HEPES. All bath solutions were adjusted to pH 7.8 and had an osmolarity of 230 mOsmol/l.

### Chromatography and mass spectrometry of animal breeding water

Solid-phase chromatography was performed on tadpole and adult frog breeding water (collection as described above), according to a previously employed method for steroids extraction from urine samples (Shackleton and Whitney, 1980; Nodari et al., 2008). Briefly, a Sep-pak C-18 column (Waters Corp) was primed with one volume methanol, followed by one volume 20% methanol/2% acetic acid. After washing with two volumes water, 100 ml of sample (either tadpole or adult frog breeding water) were passed through the column with a syringe, at a flow rate of ~20 ml/min. Then, one volume 20% methanol/2% acetic acid was passed though the column, followed by 2 ml methanol elution to recover the steroids and steroid conjugates. An API 3000 Triple Quad Mass Spectrometer with an ESI spray ion source in a negative ion mode was then used to analyze the C-18 extracts from adult and tadpole breeding water. The methanol extract was infused at 10 μl/min into the ESI source by syringe pump. The electrospray needle was set at 4.5 kV and the ion source temperature was 180°C. Nitrogen was used as the collision gas. Mass spectrum was collected by precursor ion scan and the collision energy was set at 45 eV for the collision cell (Q2).

## Results

### Sulfated steroids activate sensory neurons in the main and the vomeronasal epithelium of *Xenopus laevis*

**Figure 1:**
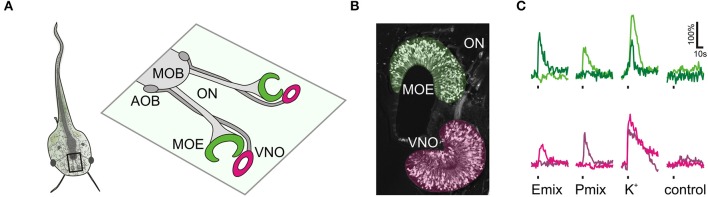
**Sulfated steroid-induced calcium responses in the olfactory organ**. **(A)** Schematic of a larval *Xenopus laevis* (stage: 52–53). The black rectangle outlines the olfactory system. An enlargement of the olfactory system is shown on the right hand side. The main olfactory epithelium (MOE) and the vomeronasal organ (VNO) are connected via the olfactory nerve (ON) to the main olfactory bulb (MOB) and the accessory olfactory bulb (AOB), respectively. **(B)** Olfactory organ of larval *Xenopus laevis* visualized by biocytin-streptavidin retrograde labeling of sensory neurons (MOE, green; VNO, magenta). **(C)** Time courses of sulfated steroid-induced [Ca^2+^]_*i*_ transients of individual sensory neurons of the MOE (green traces, 2 different cells) and the VNO (magenta traces, 2 different cells). We recorded a total of 90 P mix-responsive sensory neurons in the MOE (18 slices) and 75 in the VNO (33 slices). We recorded a total of 30 E mix-responsive sensory neurons in the MOE (18 slices) and 5 in the VNO (32 slices). Sulfated steroid mixtures were applied at a concentration of 100–200 μM. All cells responded to high K^+^ solution, but did not respond upon application of standard bath solution.

**Figure 2:**
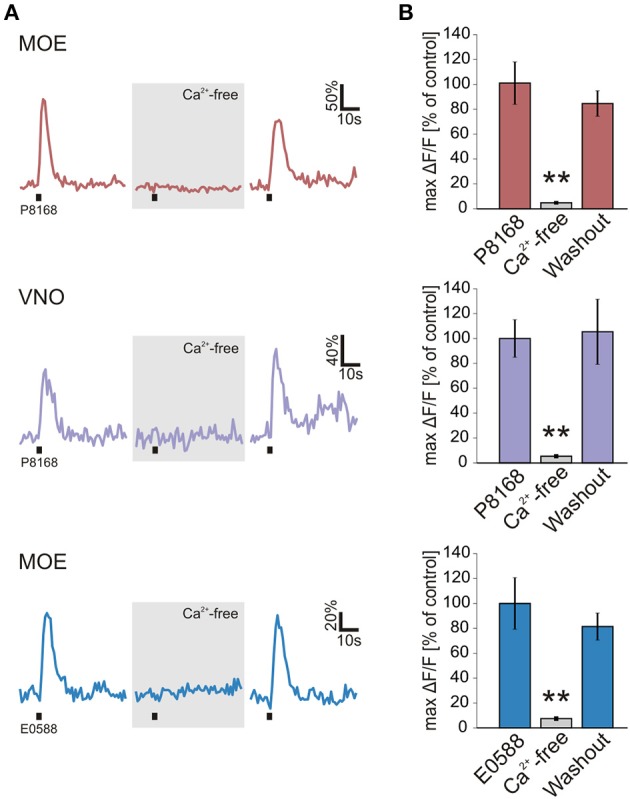
**Sulfated steroid-induced calcium transients depend on extracellular Ca^2+^**. **(A)** Calcium responses of individual neurons to single sulfated steroids (200 μM) are shown. All tested neurons, in both MOE and VNO, showed no response after 3 min incubation in Ca^2+^-free bath solution. After 3 min of washout, all responses recovered. **(B)** Mean calcium responses (±SEM), expressed as percentage of control (MOE/P8168, 9 cells, 2 slices; VNO/P8168, 9 cells, 2 slices; MOE/E0588, 9 cells, 6 slices) are shown in standard (colored columns) and Ca^2+^-free bath solution (gray columns; 3 min after application of Ca^2+^-free bath solution). All responses were virtually abolished in Ca^2+^-free bath solution (^**^p < 0.01; paired Student's *t*-test).

**Figure 3:**
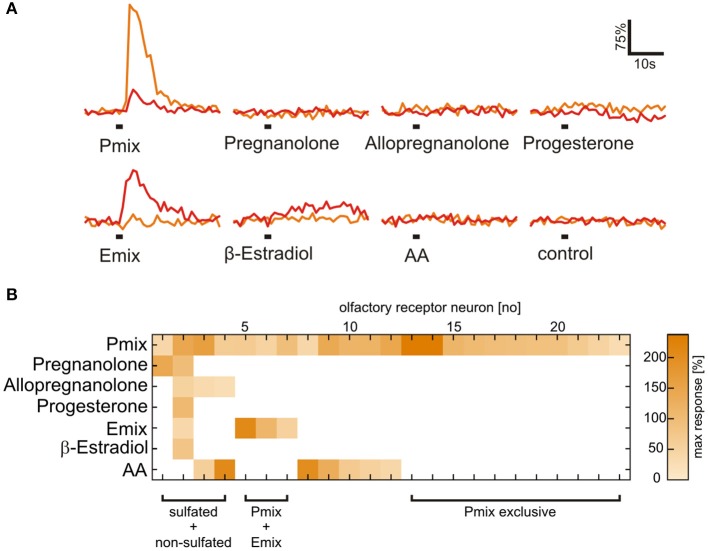
**Presence of a sulfate group is necessary for activation of most sulfated steroid-sensitive neurons**. **(A)** Sulfated steroid-induced calcium responses of two neurons in the MOE (orange and red trace). Both neurons responded upon application of E and P sulfated steroids (200 μM), but were not sensitive to their non-sulfated analogs (200 μM). **(B)** Response matrix of MOE neurons sensitive to sulfated (E mix and P mix) and non-sulfated steroids (pregnanolone, allopregnanolone, progesterone, and β-estradiol; 23 cells, 2 slices). The majority of sulfated steroids-sensitive neurons (19 cells) did not respond upon application of non-sulfated steroids. Response intensity is coded by a color gradient. A mixture of amino acids (AA, 100 μM) was applied as a control for slice viability.

Sulfated steroids from the androgen, estrogen, pregnanolone and glucocorticoid families, potent murine vomeronasal ligands (see Supplementary Table 1 and Nodari et al., 2008; Isogai et al., 2011; Celsi et al., 2012), were tested for their chemosensory activity in larval *Xenopus laevis*. Responses were measured as stimulus-induced calcium increases of sensory neurons in acute slice preparations of the olfactory organ (Figures 1A,B). We found steroid-responsive sensory neurons in the VNO and the MOE (Figure 1C). Neurons responded to pregnanolone-derived (P mix) and estrogen-derived (E mix) steroids (for more information see Materials and Methods). Thereby, in both epithelia P mix responses were more frequent than E mix responses. None of the tested androgen-derived and glucocorticoid-derived steroids were effective (not shown). All cells that responded to sulfated steroids (VNO and MOE) also showed calcium transients upon stimulation with high K^+^ bath solution, indicating that they were sensory neurons (Dittrich et al., 2014). In both epithelia, sulfated steroid-induced calcium responses were dependent on the presence of extracellular Ca^2+^, indicating that the stimulus-induced intracellular Ca^2+^ increase is mainly due to Ca^2+^ entry through plasma membrane ion channels (Figure 2). Non-sulfated analogs of the employed steroids in most cases (>80%) failed to activate sulfated-steroid-responsive neurons (Figure 3).

### Olfactory responses to sulfated steroids are conveyed and processed in the olfactory bulb

**Figure 4:**
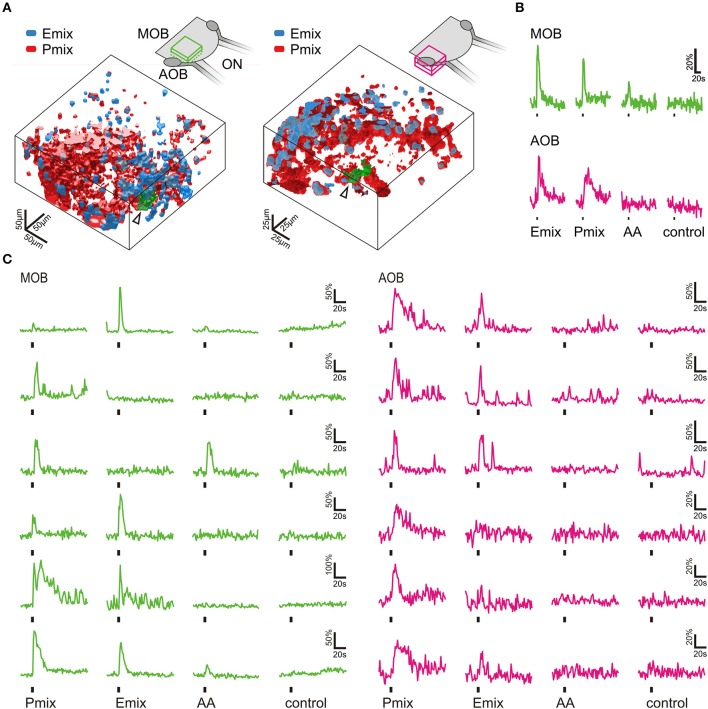
**Sulfated steroids are processed in the olfactory bulb**. **(A)** 3D rendering of reactive volumes in the MOB (left hand side) and AOB (right hand side) upon mucosal application of E and P mix (E mix-responsive areas, blue; P mix-responsive areas, red). Active regions always contained both cell somata and glomerular structures. Schematics of the olfactory bulb in the upper part show the approximate location of the responsive regions. **(B)** Calcium responses of the glomerular regions indicated by an arrow and green color in A (MOB, green traces; AOB, magenta traces). Amino acid mixture (AA, 100 μM) was applied as a positive control for activation of the MOB. **(C)** Representative calcium responses of mitral/tufted cells in the MOB (green) and AOB (magenta) upon mucosal application of sulfated steroids (E mix and P mix, 200 μM) and amino acids (AA, 100 μM). In both main and accessory olfactory bulb, mitral/tufted cells were activated by either E or P steroids, or both stimuli. Amino acids only elicited calcium responses in mitral/tufted cells of the MOB. Similar results were obtained in 4 MOBs and 3 AOBs.

To gain information about the processing of sulfated steroids in the first relay center of the system, we recorded odorant-induced calcium responses in whole mounts of the olfactory system. Nasal application of P and E mix sulfated steroids elicited transient increases in intracellular Ca^2+^ concentration in the main and accessory olfactory bulb. We detected responses in glomerular tufts and in somata of mitral/tufted cells (Figures 4A–C). Glomerular responses to the E mix were strongly overlapping with the P mix-responsive areas, but we also found regions reacting individually to each mixture (Figure 4A). Amino acid-evoked response patterns in the main olfactory bulb were also in part overlapping with the steroid-reactive areas (Figure 4B). Amino acids did not evoke any activity in the accessory olfactory bulb (Figures 4B,C). Signals detected in individual cell somata of the olfactory bulb showed various reactivity patterns to the different odorant stimuli (Figure 4C). Transection of the olfactory nerve abolished all olfactory bulb responses to odorant stimulation (data not shown).

### Tuning properties of sulfated steroid sensitive neurons

**Figure 5:**
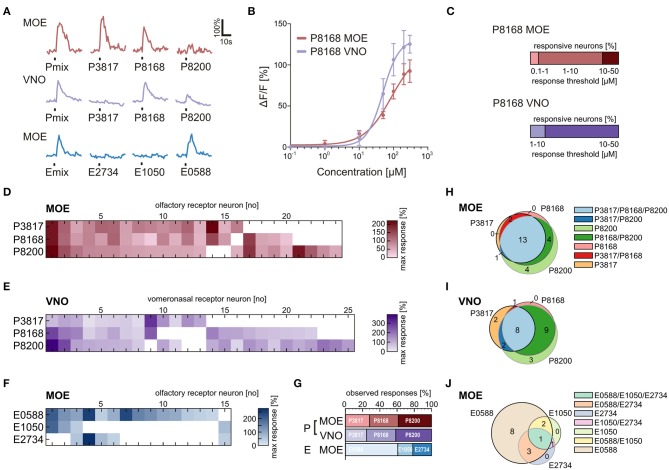
**Physiological characterization of sulfated steroid responses**. **(A)** Calcium responses of three sensory neurons upon application of sulfated steroid mixtures and of single compounds from each mixture (200 μM). **(B)** Concentration-dependent response curves for a single component of the P mix (P8168) in sensory neurons of the MOE (9 cells) and the VNO (8 cells). **(C)** Graphs showing the percentage of responsive neurons having a certain detection threshold. MOE: 0.1–1 μM, 1 cell; 1–10 μM, 8 cells; 10–50 μM, 2 cells (8 slices). VNO: 1–10 μM, 2 cells; 10–50 μM, 10 cells (6 slices). **(D)** Response profiles of P steroid-sensitive neurons in the MOE (24 cells, 7 slices; response intensities are coded by a color gradient). **(E)** Response profiles of P steroid-sensitive neurons in the VNO (25 cells, 5 slices). **(F)** Response profiles of E steroid-sensitive neurons in the MOE (15 cells, 7 slices). **(G)** Percentage of observed responses to individual sulfated steroids. P steroids showed a similar trend between MOE and VNO, with all three steroids eliciting between 20 and 40% of the total responses. A different trend was detected for E steroids, with one compound of the mixture (E0588) eliciting more than half (~60%) of the observed responses. **(H)** Venn diagram showing groups of P mix responsive neurons in the MOE. The majority of the neurons (13 cells) responded to all three components of the mixture. **(I)** Venn diagram showing groups of P mix responsive neurons in the VNO. The two main groups (9 and 8 cells) include neurons responding to two and three chemicals of the P mix. **(J)** Venn diagram showing groups of E mix responsive neurons in the MOE. The largest group (8 cells) contains neurons responding to only one chemical in the mixture, namely E0588.

We applied the individual components of the P and E mix (see Materials and Methods and Supplementary Table 1) in addition to the mixtures to obtain response profiles of individual neurons of the VNO and the MOE (Figure 5A). Similar responses for the P mix sulfated steroids were obtained in cells of the MOE and the VNO, i.e., in both epithelia most sensory neurons responded to more than one compound of the mixture. This suggests that sensory neurons in both olfactory subsystems are broadly tuned to P mix sulfated steroids (Figures 5D,E). None of the P mix compounds was detected with much higher frequency than the others (Figure 5G). In contrast, the response profiles for E mix steroids in the MOE showed a different trend. One specific compound, i.e., E0588, activated the large majority of cells. The other two compounds were less effective (Figures 5F,G). The Venn diagrams in Figures 5H–J emphasize the above findings. Overall, the average number of P and E mix-sensitive cells in the MOE and the VNO varied considerably (MOE: 5 P mix cells vs. 1.7 E mix cells/slice; VNO: 2.3 P mix cells vs. 0.16 E mix cells/slice; for absolute numbers see Figure 1). E mix-responsive cells in the VNO were too rare (5 cells in 32 slices) to perform a systematic analysis of exact response profiles.

### Sensory neurons in the main and the vomeronasal epithelium respond to the same sulfated steroid with different sensitivity

To gain insight in possible sensitivity differences to sulfated steroids in sensory neurons of the VNO and MOE, we determined dose-response curves of individual cells for P8168, a single steroid of the P mix. The recorded responses to P8168 were concentration dependent and the dose-response curves were fitted by the Hill equation (Figure 5B). The calculated EC_50_ values and Hill slopes for the two epithelia were slightly different (EC_50_: MOE, 86 μM, VNO, 49 μM; Hill slope: MOE, 1.10, VNO, 1.87). The detection thresholds, i.e., the sensitivity, for P8168 of individual sensory neurons in the MOE and the VNO were significantly different (Figure 5C; Chi-squared test, ^**^p < 0.01). On average, sensory neurons in the MOE were more sensitive to P8168 than those in the VNO. Specifically, in the MOE the majority of the tested sensory neurons had a threshold in the range of 1–10 μM, whereas the majority of VNO neurons had thresholds in the range of 10–50 μM (for exact numbers see legend of Figure 5).

### Chromatography and mass spectrometry reveal the presence of sulfated compounds in *Xenopus breeding water*

**Figure 6:**
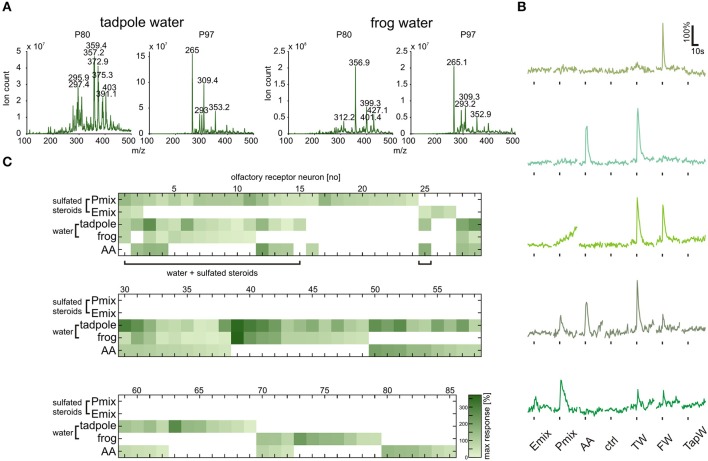
**Tadpole and frog breeding water contains sulfated compounds and activates neurons sensitive to synthetic steroids**. **(A)** Following chromatographic extraction of steroids from breeding water, precursor ion scan mass spectrometry was used to analyze the abundance of peaks that fragment to produce negative ions with m/z of 80 (${\text{SO}}_{3}^{-}$, left column) or 97 (${\text{HSO}}_{4}^{-}$, right column). **(B)** Representative calcium responses of five MOE neurons. The shown cells include the main subgroups of responding cells, i.e., cells responding to both synthetic steroids (200 μM) and breeding water (1:1 dilution), cells responding to amino acids (100 μM) and breeding water, cells responding to breeding water only, and cells responding to synthetic steroids, breeding water and amino acids. **(C)** Response matrix of neurons in the MOE responding to breeding water (1:1 dilution) and synthetic sulfated steroids (200 μM; 85 cells, 5 slices). The majority of the neurons responding to synthetic steroids also responded upon application of breeding water (16 out of 27 cells). Response intensity is coded by a color gradient. A mixture of amino acids (AA, 100 μM) was applied as a control for slice viability. TW, tadpole breeding water; FW, frog breeding water; TapW, tap water control; ctrl, bath solution control.

Sulfated steroids were shown to be excreted in mouse urine and to activate receptor neurons of the VNO (Hsu et al., 2008; Nodari et al., 2008). To obtain some insight on whether *Xenopus laevis* also excretes sulfated steroids, we performed steroid extraction from tadpole and adult frog breeding water via solid-phase chromatography. A well-established protocol for steroid extraction from urine samples (Shackleton and Whitney, 1980; Nodari et al., 2008) was employed to recover and concentrate free steroids and steroid conjugates contained in the breeding water. These extracts were then analyzed by precursor ion mass spectrometry (see Materials and Methods) for ions that could dissociate to produce fragments with a mass-to-charge ratio (*m/z*) of 80 or 97, signatures of ${\text{HSO}}_{4}^{-}$ and ${\text{SO}}_{3}^{-}$ ions produced by many sulfated steroids (Gaskell, 1988; Weidolf et al., 1988; Yan et al., 2014). This analysis revealed the presence of multiple sulfated compounds (Figure 6A), of which several have *m/z* (in the approximate range 350–450 Daltons) consistent with sulfated steroids.

### Tadpole and frog breeding water activate a large subset of sulfated steroid-sensitive sensory neurons

The fact that sulfated compounds are excreted by *Xenopus* led us to the assumption that these compounds, including endogenous sulfated steroids, could be the natural stimuli for sensory neurons activated by synthetic sulfated steroids (see Figures 1, 5). We collected tadpole and adult frog breeding water (see Materials and Methods), diluted it 1:1 in bath solution, and tested it as a stimulus in calcium imaging experiments in acute slices of the MOE. We recorded responses of 85 sensory neurons upon application of P mix steroids, E mix steroids, tadpole breeding water, adult frog breeding water, and a mixture of amino acids (Figures 6B,C). We then sorted the sensory neurons in main classes according to their response profile (Figure 6C). We identified a highly heterogeneous population of sensory neurons with different selectivity for the presented stimuli (example responses are shown in Figure 6B). Sixty-eight sensory neurons were sensitive to breeding water (only tadpole water: 26 neurons; only adult water: 10 neurons; both: 32 neurons). One fourth of these cells responded also to synthetic sulfated steroids. Remarkably, the majority of sulfated steroid-sensitive neurons were also activated by breeding water. This supports our hypothesis that sulfated steroids are excreted by *Xenopus laevis* tadpoles and frogs, similar as in mice (Hsu et al., 2008; Nodari et al., 2008). Fifty-two sensory neurons were sensitive to breeding water, but not to synthetic steroids (only tadpole water: 20 neurons; only adult water: 10 neurons; both: 22 neurons). Thirty-four out of 68 breeding water-sensitive sensory neurons responded also to amino acids, suggesting the presence of amino acids in breeding water. However, the fact that a subgroup of sensory neurons exclusively responded to breeding water suggests that additional chemosensory active compounds are excreted by *Xenopus*.

## Discussion

Sulfated steroids are urinary excretion products known to act as natural vomeronasal ligands in mice (Nodari et al., 2008; Isogai et al., 2011; Celsi et al., 2012). They account for up to 80% of the VNO neuronal responses by female urine and are thought to signal information about the sex and physiological status (Nodari et al., 2008). The present study now demonstrates that sulfated steroids are also detected by the olfactory system of *Xenopus laevis*. Interestingly, sulfated steroid are sensed and processed by both the main and the vomeronasal system in *Xenopus*, providing an unprecedented evidence of parallel processing of the same olfactory cues in two amphibian olfactory subsystems. Moreover, we found that *Xenopus* excretions contain sulfated compounds that activate a large subset of sulfated steroid-sensitive sensory neurons. This finding supports the idea that sulfated steroids could mediate intraspecific olfactory communication in amphibians.

It has long been assumed that the main and the vomeronasal systems are sensitive to different sets of chemosensory signals. While the main system was thought to solely detect common odors, the vomeronasal system was believed to specifically detect pheromones (Liberles, 2014; but see Hudson and Distel, 1986). Studies in rodents have reported that both systems respond to overlapping sets of stimuli (Sam et al., 2001; Spehr et al., 2006b; Zufall and Leinders-Zufall, 2007). Common odors as well as pheromones have been reported to activate both systems (Leinders-Zufall et al., 2004; Xu et al., 2005; Spehr et al., 2006a). The present study shows that the same set of molecular cues, i.e., sulfated steroids, are detected by two olfactory subsystems in an amphibian species. The frequency of P and E mix-sensitive cells in the MOE and the VNO of *Xenopus* varies considerably. In both epithelia P mix-sensitive cells were more frequent than E mix-sensitive cells. Especially in the VNO the E mix-sensitive cells are a rare subpopulation. These differences are reflected also in the olfactory bulb responses. Mucosal application of P mix activated larger bulb areas than the E mix in both the main and the accessory olfactory bulb (Figure 4A). Furthermore, response profile analysis for the single components of the mixtures highlighted differences in tuning properties of individual neurons (see Figures 5D–J). Sensory neurons in both epithelia were broadly tuned to all single components of the P mix. E mix-sensitive cells in the MOE were rather narrowly tuned to one component of the mixture (E0588). The low frequency of E mix-sensitive neurons in the VNO prevented the recording of response profiles to steroids of this group. The low selectivity of the P mix neurons might be due to high structural similarity among the mixture components. A similar broad tuning of individual P steroid-sensitive VNO neurons has been reported in mouse (Nodari et al., 2008; Meeks et al., 2010; Turaga and Holy, 2012). Interestingly, these findings are in contrast with results of another study with isolated mice VNO neurons, where all P sulfated steroid-sensitive vomeronasal neurons were narrowly tuned to one steroid (Celsi et al., 2012). E mix-responsive VNO neurons are rather narrowly tuned also in mice (Nodari et al., 2008).

The dose-response curves and threshold values for P8168 (VNO and MOE) obtained in *Xenopus* were in the same range as those obtained in VNO neurons of mice (Nodari et al., 2008; Celsi et al., 2012). Rather unexpectedly the thresholds in the MOE of *Xenopus* were significantly lower than in the VNO. These differences in sensitivity could indicate that the activation of both systems is not just redundant, but rather signal different information, possibly triggering different behavioral responses. A similar situation is present in the mouse olfactory system. Major histocompatibility complex peptides, chemosensory signals that carry information about individuality (Leinders-Zufall et al., 2004; Spehr et al., 2006a), mediate different behavioral responses via the main and accessory system (Kelliher, 2007).

In *Xenopus laevis*, strikingly different than in later diverging vertebrates such as rodents, vomeronasal type-1 and more “ancestral” vomeronasal type-2 receptor genes are expressed in the MOE (Date-Ito et al., 2008; Gliem et al., 2013; Syed et al., 2013), together with OR-type and trace amine associated receptor genes (Mezler et al., 1999; Gliem et al., 2013). Sensory neurons of the *Xenopus* VNO exclusively express later diverging vomeronasal type-2 receptor genes (Hagino-Yamagishi et al., 2004; Syed et al., 2013). This allows one to speculate about the receptor gene families involved in the detection of sulfated steroids in the two olfactory subsystems of *Xenopus*. It appears very likely that the steroid responses in the VNO are mediated by vomeronasal type-2 receptors. This receptor family has been reported to be involved in the detection of P steroid responses in the rodent accessory olfactory system (Hammen et al., 2014). However, the MOE responses could be generated by any of the receptor families expressed in this epithelium. In the mouse VNO, E steroids have been shown to activate vomeronasal type-1 receptors (Isogai et al., 2011), therefore it appears plausible that at least some responses in the *Xenopus* MOE are mediated by this receptor family. Together, the available data about receptor expression in the two epithelia of *Xenopus* suggests that different sets of receptors are involved in the steroid detection in the MOE and the VNO. This is supported also by the differences in the dose-response curves and the different thresholds for P8168 in the two epithelia reported in the present study. Our results show that sulfated steroids appear to be a class of conserved chemosensory stimuli in the tetrapod lineage. The evolutionary relevance of these stimuli is further supported by the fact that sulfated steroids (though different than those used in the present study) serve as migratory pheromones in lamprey, a primitive jawless fish (Sorensen et al., 2005). The information about intraspecific chemical communication in adult anurans is very limited, and virtually nothing is known for larval anurans (Belanger and Corkum, 2009; Houck, 2009; Poth et al., 2012; Woodley, 2014). The presence of sulfated compounds, possibly sulfated steroids, in excretion products of larval and adult *Xenopus* indicates that they could serve as intraspecific communication cues. The fact that the majority of neurons sensitive to synthetic sulfated steroids also respond to breeding water is certainly in line with this speculation.

## Conclusions

The available functional data on the amphibian accessory olfactory system is very scarce if compared to mammals. In particular, a physiological characterization of vomeronasal responses in anuran amphibians was never performed, due to the lack of known stimuli. In this study, we identified sulfated steroids as a novel class of amphibian olfactory stimuli, which also activate the vomeronasal organ. Sulfated steroids are known to function as migratory pheromones in lamprey and have recently been identified as the predominant vomeronasal ligands in female mouse urine. In mice they are thought to transmit information about the animal's physiological status, although a clear behavioral output has not been identified yet. Our data show that sulfated steroids are potent stimuli of both the main and the vomeronasal system of the amphibian *Xenopus laevis*. Moreover, breeding water containing sulfated compounds, with mass-to-charge ratios consistent with sulfated steroids, activated a large subset of steroid-sensitive sensory neurons, suggesting their involvement in amphibian intraspecific communication. Taken together, our study shows that sulfated steroids are conserved vomeronasal stimuli functioning in phylogenetically distant classes of tetrapods living in aquatic and terrestrial habitats. Future studies will be necessary to elucidate whether sulfated steroid responses in fact trigger behavioral responses in larval (and adult) *Xenopus*, and if the different information channels (main and accessory olfactory system) elicit diverse behaviors.

## Author contributions

The experiments were conceived and designed by AS, TH, and IM. The experiments were performed by AS, TO and XF. AS, TH, TO, XF, and TEH analyzed the data. AS, TH, TEH and IM wrote the paper. All authors participated in the discussion of the data and in production of the final version of the manuscript.

### Conflict of interest statement

The authors declare that the research was conducted in the absence of any commercial or financial relationships that could be construed as a potential conflict of interest.

## Abbreviations

MOE: main olfactory epithelium
VNO: vomeronasal organ.
