# Supplementary material for: Dual processing of sulfated steroids in the olfactory system of an anuran amphibian
Source: Front Cell Neurosci. 2015 Sep 23;9:373. doi: 10.3389/fncel.2015.00373 (PMC4585043; doi:10.3389/fncel.2015.00373)
Supplement: Supplementary file 1 [file Table1.PDF]

Table 1. List of sulfated steroids (sodium salts)

Catalogue ID	Chemical name	Trivial name	Chemical structure
**A2398**	5α-ANDROSTAN-3β-OL-16-ONE SULPHATE	–	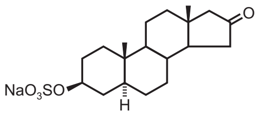
**A6940**	4-ANDROSTEN-17α-OL-3-ONE SULPHATE	EPITESTOSTERONE SULPHATE	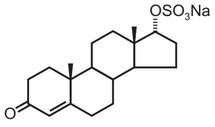
**E0588**	1, 3, 5(10), 7-ESTRATETRAEN-3, 17β-DIOL 3-SULPHATE	EQUILOL SULPHATE	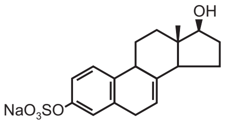
**E1050**	1, 3, 5(10)-ESTRATRIEN-3, 17β-DIOL DISULPHATE	17β-ESTRADIOL SULPHATE	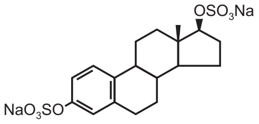
**E2734**	1, 3, 5(10)-ESTRATRIEN-3, 16α, 17β-TRIOL 17-SULPHATE	ESTRIOL 17-SULPHATE	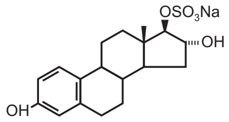
**P3817**	5α-PREGNAN-3α-OL-20-ONE SULPHATE	ALLOPREGNANOLONE SULPHATE	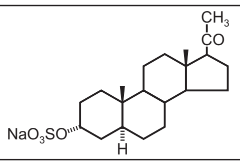
**P8168**	5β-PREGNAN-3α-OL-20-ONE SULPHATE	PREGNANOLONE SULPHATE	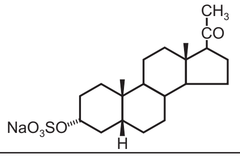
**P8200**	5β-PREGNAN-3β-OL-20-ONE SULPHATE	EPIPREGNANOLONE SULPHATE	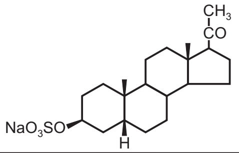
**Q1570**	4-PREGNEN-11β, 21-DIOL-3, 20-DIONE 21-SULPHATE	CORTICOSTERONE 21-SULPHATE	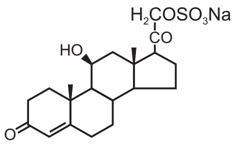
**Q2525**	4-PREGNEN-17, 21-DIOL-3, 11, 20-TRIONE 21-SULPHATE	CORTISONE 21-SULPHATE	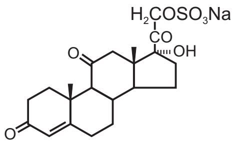
**Q3910**	4-PREGNEN-11β, 17, 21-TRIOL-3, 20-DIONE 21-SULPHATE	HYDROCORTISONE 21-SULPHATE	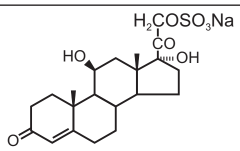

Catalogue ID codes are from Steraloids (Newport, USA), and code prefixes refer to the steroid family (A, androgen; E, estrogen; P, pregnanolone; Q, glucocorticoid).
